# Behavioral Outcomes Differ between Rotational Acceleration and Blast Mechanisms of Mild Traumatic Brain Injury

**DOI:** 10.3389/fneur.2016.00031

**Published:** 2016-03-14

**Authors:** Brian D. Stemper, Alok S. Shah, Matthew D. Budde, Christopher M. Olsen, Aleksandra Glavaski-Joksimovic, Shekar N. Kurpad, Michael McCrea, Frank A. Pintar

**Affiliations:** ^1^Department of Neurosurgery, Medical College of Wisconsin, Milwaukee, WI, USA; ^2^Clement J. Zablocki Veterans Affairs Medical Center, Milwaukee, WI, USA; ^3^Neuroscience Research Center, Medical College of Wisconsin, Milwaukee, WI, USA; ^4^Department of Pharmacology and Toxicology, Medical College of Wisconsin, Milwaukee, WI, USA

**Keywords:** concussion, rotational acceleration, blast injuries, mechanism of injury, biomechanics

## Abstract

Mild traumatic brain injury (mTBI) can result from a number of mechanisms, including blunt impact, head rotational acceleration, exposure to blast, and penetration of projectiles. Mechanism is likely to influence the type, severity, and chronicity of outcomes. The objective of this study was to determine differences in the severity and time course of behavioral outcomes following blast and rotational mTBI. The Medical College of Wisconsin (MCW) Rotational Injury model and a shock tube model of primary blast injury were used to induce mTBI in rats and behavioral assessments were conducted within the first week, as well as 30 and 60 days following injury. Acute recovery time demonstrated similar increases over protocol-matched shams, indicating acute injury severity equivalence between the two mechanisms. Post-injury behavior in the elevated plus maze demonstrated differing trends, with rotationally injured rats acutely demonstrating greater activity, whereas blast-injured rats had decreased activity that developed at chronic time points. Similarly, blast-injured rats demonstrated trends associated with cognitive deficits that were not apparent following rotational injuries. These findings demonstrate that rotational and blast injury result in behavioral changes with different qualitative and temporal manifestations. Whereas rotational injury was characterized by a rapidly emerging phenotype consistent with behavioral disinhibition, blast injury was associated with emotional and cognitive differences that were not evident acutely, but developed later, with an anxiety-like phenotype still present in injured animals at our most chronic measurements.

## Introduction

Mild traumatic brain injury (mTBI) can be caused by a number of mechanisms, including blunt impact, head rotational acceleration, exposure to blast, and penetration of projectiles (1, 2). In addition to the severity of injury and patient characteristics, the mechanism of injury is likely to be an important factor in the acute and chronic clinical profile of the patient. Recent clinical studies have begun to address the influence of injury mechanism, comparing outcomes between veterans with blast-related traumatic brain injury to those with non-blast etiologies, such as falls, motor vehicle accidents, and assaults. Those studies reported a unique clinical profile for veterans that have sustained blast injury, indicating that penetrating brain injuries, facial injuries, and post-traumatic stress disorder (PTSD) symptoms were more common than in patients with non-blast TBI (3). However, effects of injury mechanism on the neuropsychological profile of the patient are less clear. A recent review of the clinical literature indicated that individuals who sustain mTBI (i.e., concussion) resulting from blast were somewhat more likely to develop trauma symptoms and other emotional difficulties than individuals with non-blast concussion, although the effect size was limited (4). As an example, Lippa and colleagues indicated higher post-traumatic stress symptoms for veterans reporting blast TBI than those reporting non-blast TBI (5). However, the authors acknowledged that there were no differences in post-concussive symptom severity or symptom cluster based on mechanism. Unlike changes in emotionality, there appears to be limited evidence to support differences in cognitive outcomes based on concussive mechanism. For example, Belanger and colleagues indicated that there was no strong evidence that blast was different from other mechanisms with regard to cognitive sequelae on select measures (6). Limited clinical evidence to support mechanism-based outcomes may indicate a lack of actual differences or may be attributed, at least in part, to variability in exposure characteristics and patient histories.

Different clinical outcomes associated with unique spatial distribution and pathologies would be expected, given the significant differences in injury mechanism between blast and non-blast TBI. Although the exact mechanism of tissue damage remains controversial, theories on the mechanism of blast TBI have included skull flexure (7), thoracic mechanisms (8, 9), and cerebrospinal fluid cavitation (10). Other studies have focused on the interaction of the shock wave with brain tissues, resulting in brain tissue shear stresses (11). This mechanism is particularly relevant, given that our group has shown in a post-mortem human subject model that shock wave overpressure enters the cranium and interacts with the intracranial contents with the Friedlander waveform essentially intact, although the magnitude is somewhat attenuated (12). Once inside the cranium, the shock wave can interact with brain tissues on a local level to produce extensive neuronal death or altered neuronal function, loss of glial cells, and astrocytosis (13, 14). Behavioral outcomes were shown in a rodent model to vary with peak shock wave overpressure magnitude when the head was constrained against significant rotations and the body was protected (15). This overpressure-induced phenomenon differs from the traditional rotational acceleration-induced mechanism that produces strains in the brain tissues through inertia (16, 17) and anatomical inhomogeneities (18). Characteristics of the head rotational acceleration pulse (i.e., magnitude and duration) were shown to influence regionally dependent strains within the brain tissues in a computational model (19) and behavioral outcomes in a rodent model (20). Likewise, mechanical tolerance for concussion is dependent on the rotational acceleration characteristics (21). Therefore, it can be hypothesized that differing outcomes are expected from these two mechanically distinct phenomena.

As mentioned, a major reason that clinical studies may not show remarkable differences between blast and non-blast TBI is the variability in injury profiles, heterogeneity of the documented mechanism, and exposure history between patients. Individuals exposed to blast often sustain additional injuries associated with secondary, tertiary, and quaternary mechanisms (22). Specifically, secondary and tertiary injuries can involve head impact with associated head acceleration/deceleration producing TBI through more conventional non-blast mechanisms. Confounding the issue is that composite clinical profiles across a population are complicated by individual variability in subconcussive exposure and concussion history. Prior concussion history has been shown to influence chronic cognitive and emotional outcomes (23, 24). Therefore, the question remains whether isolated blast exposure leads to different outcomes from non-blast mechanisms. This remains an important clinical issue, given the number of veterans exposed to single or repetitive blast injuries during the past decade (25). It also remains an important biomechanical issue, with the development of personal protective equipment to prevent or limit traumatic brain injury due to head impact/rotational acceleration or blast remaining largely in the early phases. Therefore, the objectives of the current study were to determine whether differences exist in the severity and time course of cognitive and emotional changes following blast and non-blast mechanisms of traumatic brain injury. We accomplished this using blast and rotational injury models that we previously demonstrated to result in mild injuries that are proportional in magnitude between the two mechanisms.

## Materials and Methods

The current protocol consisted of exposing rats to rotational injury, blast injury, or sham procedure followed by behavioral assessments during the first week and at 1 month or 2 months following the injury or sham procedure to characterize acute and chronic cognitive and behavioral changes associated with different injury mechanisms. All injury and behavioral testing was conducted with approval from the Institutional Animal Care and Use Committee at the Zablocki Veterans Affairs Medical Center in Milwaukee, WI, USA.

### Animals

Sprague-Dawley rats (weight: 289 ± 21 g) were used. Rats were housed individually in standard cages, maintained on a 12-h light/dark cycle, and provided free access to food and water throughout the experimental period. Separate groups of rats were used at each time point for behavioral assessments.

### Rotational Injury Procedure

**Figure 1:**
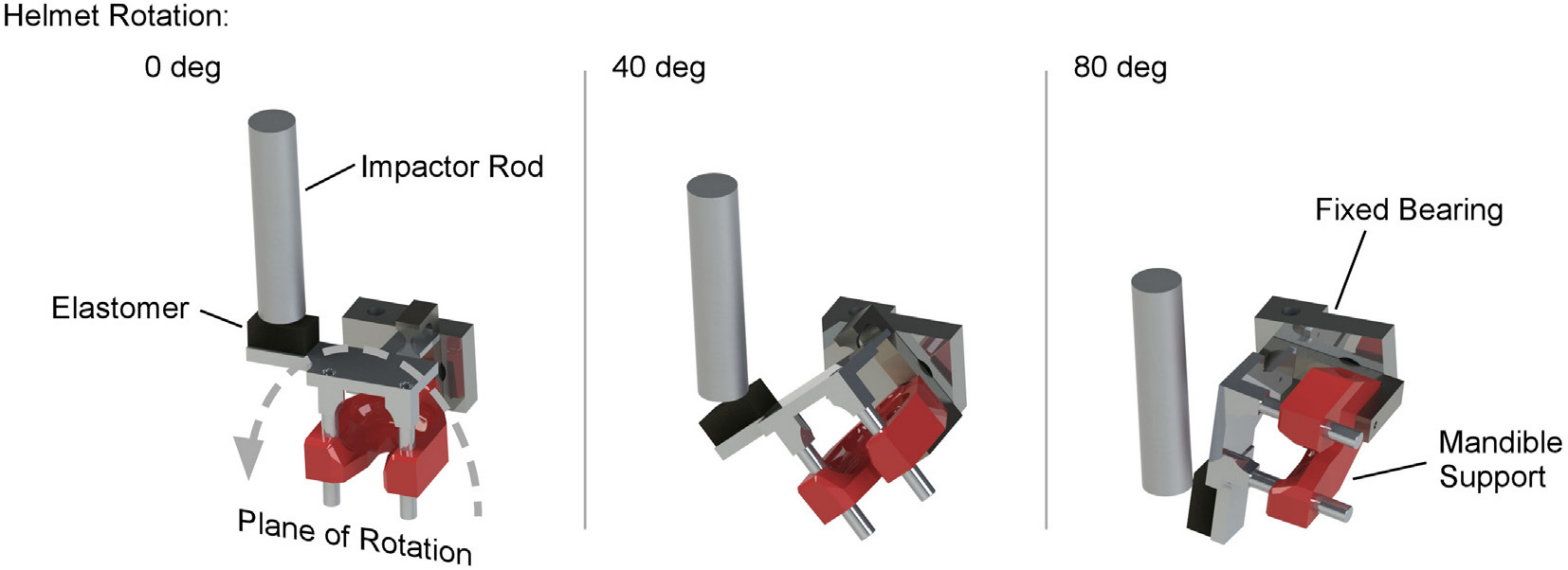
**MCW rotational injury device that induces mild traumatic brain injury in rats through pure coronal plane head rotational acceleration**. The device consists of a rodent helmet with laterally extended moment arm. An impactor rod is accelerated to impact the moment arm, causing the helmet to rotate in the coronal plane. Impact characteristics are modulated to control head rotational acceleration magnitude and duration using impactor velocity and material characteristics of an elastomer interface on the struck surface of the moment arm.

Rats were exposed to high rate head rotational acceleration using the Medical College of Wisconsin (MCW) Rotational Injury Device (Figure 1) (20). The model consists of a rat helmet with laterally extended moment arm, impactor mass, and drop tower (26). The impactor was accelerated by gravity down a drop tower to impact the moment arm and generate sufficient force to rotate the device in the coronal plane. Characteristics of the rotational acceleration versus time pulse were modulated by the mass of the impactor, initial drop height, and characteristics of the elastomer interface material between the impactor and the moment arm (27). An accelerometer attached to the distal end of the moment arm measured tangential linear acceleration, which was converted to rotational acceleration of the helmet versus time. Our previous studies and extensive testing ensured that magnitude and duration of the rotational acceleration versus time pulse were independently modulated and could be accurately measured for each exposure.

On the day of the injury procedure, rats were individually transported to the laboratory and placed in an isoflurane induction box for 5 min, where they were exposed to 4.0% isoflurane in oxygen. Plane of anesthesia was confirmed by the absence of the toe pinch reflex prior to removal from the induction box. A nose cone was then used to deliver continuous 1.5% isoflurane during placement in the rotational helmet and attachment of the helmet to the injury device. Rats were given a single dose of Carprofen (5.0 mg/kg), and anesthesia was removed immediately prior to head rotational acceleration exposure of a predetermined acceleration magnitude and duration. Following exposure to head rotational acceleration, rats were removed from the device, allowed to breathe freely, placed on a warming blanket until reappearance of the righting reflex, returned to their cages, supervised for at least 15 min, and periodically supervised until 6 h post exposure. For the current study, all rats in the rotational injury group were exposed to pulses designed to produce 350 krad/s^2^ peak head rotational acceleration with a duration of 3.5 ms. Control rats were exposed to the entire experimental protocol, including anesthesia and placement in the helmet, without head rotational acceleration.

### Blast Injury Procedure

**Figure 2:**
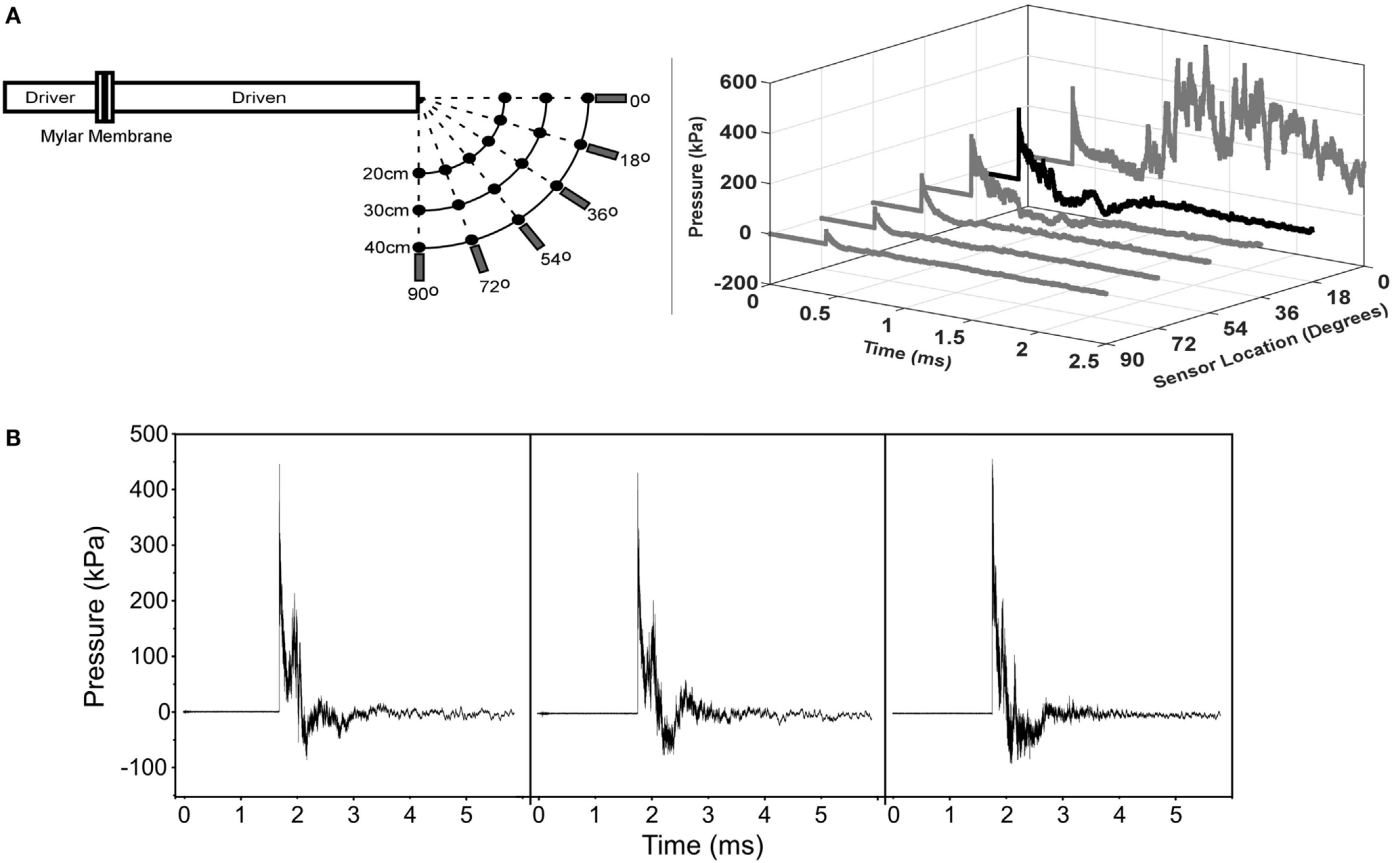
**MCW Shock Tube that produces mild traumatic brain injury in rats by off-axis exposure to shock wave overpressure**. Animals are placed 18° away from the axis of the shock tube to avoid exposure to exhaust gases than can create injury or lead to significant head rotational accelerations. **(A)** Calibration traces of the shock tube showing pressure versus time traces at different angles relative to the shock tube for the 40-cm radial location. This figure shows that exhaust gases are focused primarily radially from the exit of the tube and the effect of those gases on the overpressure trace dissipates at greater angles away from the axis. **(B)** Representative pressure traces recorded during the injury exposures for animals subjected to shock wave overpressure injury. The three traces are representative of all pressure versus time traces incorporated in this study and demonstrate the repeatability of our shock tube device. Overpressure versus time traces were recorded immediately adjacent to the head of the rat during all injury exposures.

A custom shock tube with a 3.6-cm inner diameter, 3.0-m driven section, and 0.3-m driver section was used to create shock waves with controlled overpressure magnitudes (Figure 2) (15). A mylar membrane separated driver and driven sections. The driver section was pressurized with helium until internal pressure exceeded bursting pressure of the membrane, resulting in membrane rupture. The shock tube was previously characterized and was shown to produce accurate and repeatable shock waves across a range of overpressure magnitudes (28). Pressure transducers (Piezotronics, Inc., Depew, NY, USA) were oriented to record face-on pressures at a sampling rate of 10 MHz at selected locations and were placed immediately adjacent to the head for all blast exposures.

On the day of the injury procedure, rats were individually transported to the laboratory and placed in an isoflurane induction box for 5 min, wherein they were exposed to 4.0% isoflurane in oxygen. Plane of anesthesia was confirmed by the absence of the toe pinch reflex prior to removal from the induction box. A nose cone was then used for continuous delivery of 1.5% isoflurane during placement in the decapicone and holder. The nose cone was removed immediately prior to shock wave exposure. Rats were placed 17 cm from the shock tube opening. Since the exhaust gasses, or blast wind, can lead to considerable head acceleration, animals were placed 18° lateral to the shock tube axis to limit blast wind exposure. Prior characterization of the shock tube demonstrated minimal blast wind effects at the location chosen for this study (Figure 2) (28). Moreover, the head was constrained laterally and inferiorly to prevent head rotational acceleration-induced injury (15), and all shock wave exposures were conducted with the sagittal plane of the rat head perpendicular to the radial axis from the shock tube opening. A metal cylinder was also placed around the body to limit shock wave overpressure exposure of the torso. Prior work indicated that the cylinder was effective at reducing peak overpressures to <20% of overpressure magnitudes recorded at the head. Following shock wave exposure, rats were removed from the device, allowed to breathe freely, placed on a warming blanket until reappearance of the righting reflex, returned to their cages, supervised for at least 15 min, and periodically supervised until 6 h post exposure. For the current study, all rats in the blast injury group were subjected to exposures designed to produce 450-kPa peak shock wave overpressure with a duration of 0.4 ms. Control rats were exposed to the entire experimental protocol, including anesthesia and placement in the injury device, without exposure to shock wave overpressure.

### Behavioral Assessment Protocol

Acute injury severity was assessed using recovery time, measured as the amount of time following removal of anesthesia just prior to injury or sham procedure until return of the righting reflex. Real time videos were obtained during all injury and sham procedures with specific events indicated by the animal technician. Those videos were used to measure the times of anesthesia removal, injury induction, placement on the warming blanket, and return of the righting reflex.

The elevated plus maze (EPM) assessment was used to quantify activity and emotional-type behaviors following TBI in rats. Rats underwent the EPM assessment on days 2 (acute), 30, or 60 post injury. The maze consisted of four perpendicular 10 cm × 50 cm arms suspended 82 cm above the floor. A 10 cm × 10 cm central platform connected the arms. One pair of opposing arms was enclosed by 32-cm-high walls, while the other two arms and the center platform were uncovered. Rats were initially placed on the central platform facing one of the two open arms. The animals were allowed to explore the maze for 5 min and tracked using a digital video camera mounted above the maze. Metrics quantified during the test include total distance traveled, total number of arm changes, the number of entries into and time spent in open areas (center platform and uncovered arms), and the amount of time spent in open areas/arms per entry. These metrics were automatically quantified using Ethovision computer tracking system. Behaviors associated with post-injury activity included total number of arm changes and total distance traveled. Behaviors associated with changes in emotionality included entries and time in the open areas.

The Morris water maze (MWM) Visuo-Spatial Learning Paradigm was used to grade post-traumatic anterograde amnesia and spatial learning following TBI in rats (29, 30). The MWM has been validated for these purposes and utilized in many studies investigating behavioral change as part of animal models of acquired brain injury. The paradigm consisted of 3 days of testing on post-injury days 1–3 (acute), 29–31, or 59–61. Each day of testing included a set of four trials resulting in 12 trials across 3 days. The four trials per set consisted of initially placing the rats at the four cardinal locations within the 183-cm diameter maze (N, E, S, W), facing the outer wall. During each trial, rats were allowed to swim in the 25-cm deep water until finding and mounting a 10-cm diameter hidden platform submerged 1 cm below the water surface, or until 60 s had passed. The platform was located between the cardinal axes (e.g., SE) halfway between the center and outer wall. Location remained constant for the four trials of a set, but was changed to a randomized location from set to set. Water temperature remained within 1°C of 24°C for all trials and the maze was located in a room with numerous visual cues external to the maze and oriented identically for each session. Visual cues were also placed inside the maze. A computerized tracking system and software (Ethovision V8.0, Noldus Information Technology, Wageningen, The Netherlands) recorded several metrics during each MWM trial. Latency to find the hidden platform (s) was measured for each trial and compared between injury groups and between successive trials/sets. Cognitive deficits were associated with greater latency.

### Statistical Analysis

Two-factor analysis of variance (ANOVA) was used to determine statistically significant differences (*p* < 0.05) in EPM metrics, accounting for injury group and assessment period. *Post hoc* analysis using Tukey’s method was used to determine significant differences between injury groups or assessment periods. Pairwise comparisons with correction for multiple comparisons were also conducted at each assessment period for metrics demonstrating a significant main effect of injury group or interaction. A random effects statistical model was used to predict latency to find the hidden platform in the MWM and determine significant differences based on injury group and assessment times.

## Results

A total of 128 rats were subjected to the experimental protocol, including injury or sham procedure and behavioral testing either within the first week, 30-days, or 60-days post injury. Nineteen and 24 rats were subjected to the entire blast or rotational protocol without exposure to shock wave or head rotational acceleration. Forty-six and 39 rats received shock wave or rotational acceleration injury. Including 5 min in the anesthesia induction box for all rats prior to and nose cone anesthesia during placement in the experimental device, rats receiving the rotational acceleration protocol were exposed to anesthesia for 524 ± 61 s and rats receiving the blast protocol were exposed to anesthesia for 389 ± 24 s. Rats receiving rotational injury were subjected to coronal plane head rotational acceleration with magnitude of 365 ± 31 rad/s^2^ (mean ± SD) and duration of 3.5 ± 0.4 ms. There were no significant differences in rotational acceleration characteristics between groups of rats used in the three different assessment periods. Rats receiving blast injury were subjected to shock wave exposure with 447 ± 30 kPa overpressure with 0.41 ± 0.08 ms duration and 45 ± 8 kPa × ms impulse. Shock wave impulse was consistent with prior studies investigating mTBI in both mice and organotypic hippocampal slice cultures (31, 32). There were no significant differences in shock wave characteristics between groups of rats used in the three different assessment periods. All experimental and control rats survived the anesthesia and injury procedures without skull or cervical spine fracture. Recovery time, measured as the amount of time from the injury/sham procedure until return of the righting reflex, was used to equate injury levels.

### Recovery Time

**Figure 3:**
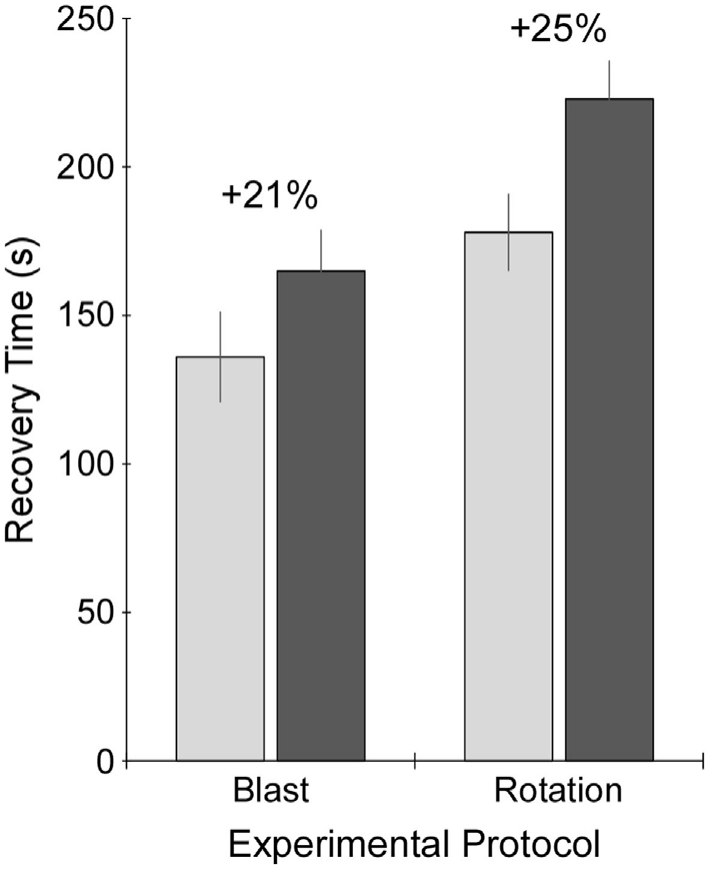
**Recovery time following exposure to sham procedure (light gray), or blast or rotational injury procedure (dark gray)**. Recovery time was measured as the time from removal of anesthesia just prior to injury or sham procedure until return of the righting reflex. Data are presented as mean plus/minus SE.

A comparison of recovery times between controls under the blast protocol and rotational protocol was first performed. Recovery times for the sham group under the rotational protocol were significantly greater (+31%) than those for the blast protocol (*p* < 0.05) (Figure 3). Therefore, recovery times for injured rats were compared to control rats that had received the same anesthesia protocol (i.e., blast or rotational). Accordingly, blast-injured rats had 21% greater recovery times than control rats receiving the blast anesthesia protocol. Likewise, rats receiving rotational injury had 25% greater recovery times than control rats receiving the rotational anesthesia protocol. Similar increases in recovery times were indicative of similar injury severities for the blast and rotational injury protocols.

### Elevated Plus Maze

Comparison of EPM activities between control rats receiving either the blast or rotational anesthesia protocols revealed no significant differences for any of the analyzed metrics including total number of arm changes, open area entries, time in the open areas, and total distance traveled for any of the analyzed time points (2, 30, and 60 days). Therefore, control rats from both protocols were grouped for comparison to injured rats.

**Figure 4:**
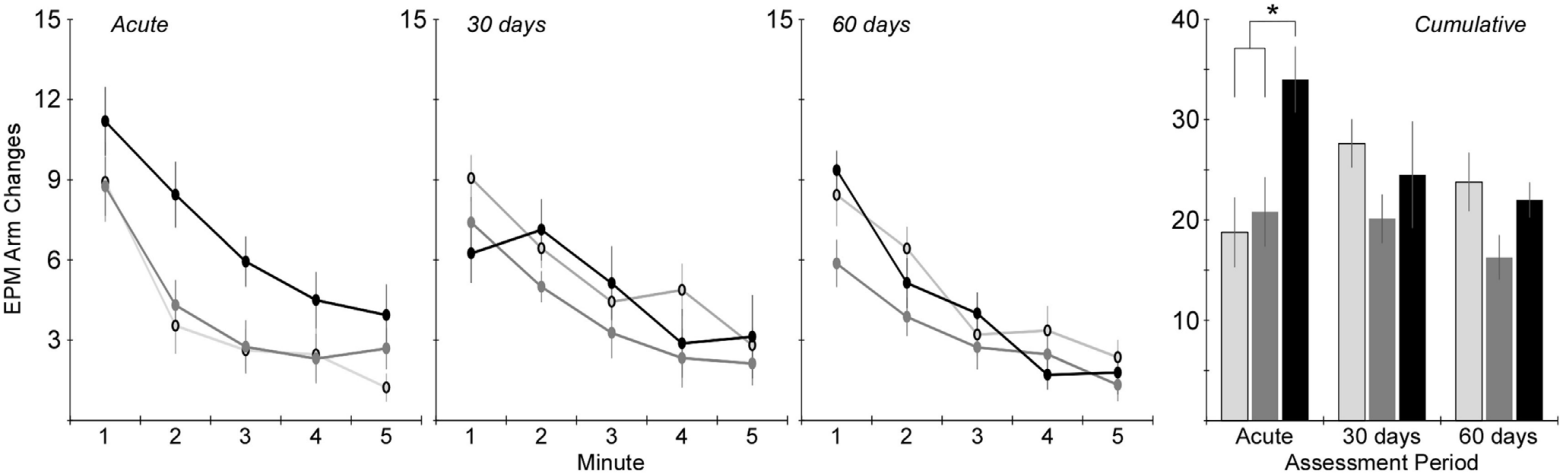
**Total number of arm changes during the 5-min elevated plus maze protocol (mean ± SE) in control rats (light gray), blast-injured rats (dark gray), and rotationally injured rats (black)**. Minute-by-minute data are presented for each of the assessment time points on the left and cumulative data across all 5 min are presented on the right. Statistically significant pairwise differences during each assessment period are indicated by an asterisk (*).

Activity in the EPM was assessed using the total number of arm changes during the 5-min trial (Figure 4). The number of rats exposed to the EPM at each time point is shown in Table 1. Two-factor ANOVA revealed that the total number of arm changes was significantly dependent on injury group (*p* < 0.005), but not assessment period. *Post hoc* assessments revealed that the blast and rotational groups were significantly different from one another. Pairwise statistical comparisons with adjustment for multiple comparisons were also conducted to determine statistically significant injury group-based differences at each assessment time. The greatest differences in number of arm changes across all groups were evident at the acute assessment time, wherein rats receiving rotational acceleration injury had an average of 61 and 79% more arm changes (*p* < 0.05) than the blast injury group and shams. At the 30-day assessment, the blast injury group demonstrated a trend of decreasing arm changes (*p* > 0.05) with an average of 20 and 29% fewer arm changes than the rotational injury group and shams. A similar trend of decreased activity (*p* > 0.05) was evident for the blast injury group at the 60-day assessment.

**Table 1:** **Elevated plus maze metrics for each of the three experimental groups at the three assessment time periods**.

	Acute			30 days			60 days		
	Controls *n* = 13	Blast *n* = 16	Rotation *n* = 16	Controls *n* = 16	Blast *n* = 15	Rotation *n* = 9	Controls *n* = 14	Blast *n* = 15	Rotation *n* = 14
Distance traveled (mm)	888 ± 144	1068 ± 92	1264 ± 93	1247 ± 142	905 ± 92	1101 ± 216	1056 ± 103	833 ± 100	991 ± 79
Open area time per entry (s)	4.5 ± 0.6	3.6 ± 0.3	3.5 ± 0.3	3.7 ± 0.3	4.0 ± 0.3	4.9 ± 0.7	4.3 ± 0.6	5.7 ± 0.7	4.4 ± 0.6
Open arm time per entry (s)	6.2 ± 1.1	7.9 ± 1.3	5.9 ± 0.7	**5.8 ± 0.9**	9.1 ± 1.4	**12.9 ± 2.4**	9.1 ± 2.5	10.5 ± 1.7	7.4 ± 1.6

*Open area time included time in the center and open arms of the maze, whereas open arm time included only time in the open arms. Statistically significant group differences were only identified for open arm time per entry at the 30-day assessment period. *Post hoc* analysis revealed significant differences (*p* < 0.01, denoted in bold) between rats receiving rotational injury and controls*.

Analysis of the total distance traveled during the 5-min EPM assessment revealed similar trends to the total number of arm changes (Table 1), although two-factor ANOVA did not identify a statistically significant influence of either injury group or assessment period (*p* > 0.05). Also similar to the number of arm changes was that the primary difference in total distance traveled between groups was evident at the acute assessment, wherein rats receiving rotational acceleration injury traveled 18 and 42% more distance than rats receiving blast injury or shams. Those differences essentially normalized by the 30- and 60-day assessments, although rats receiving blast injury demonstrated approximately 20% less distance traveled than the other groups at those time points.

**Figure 5:**
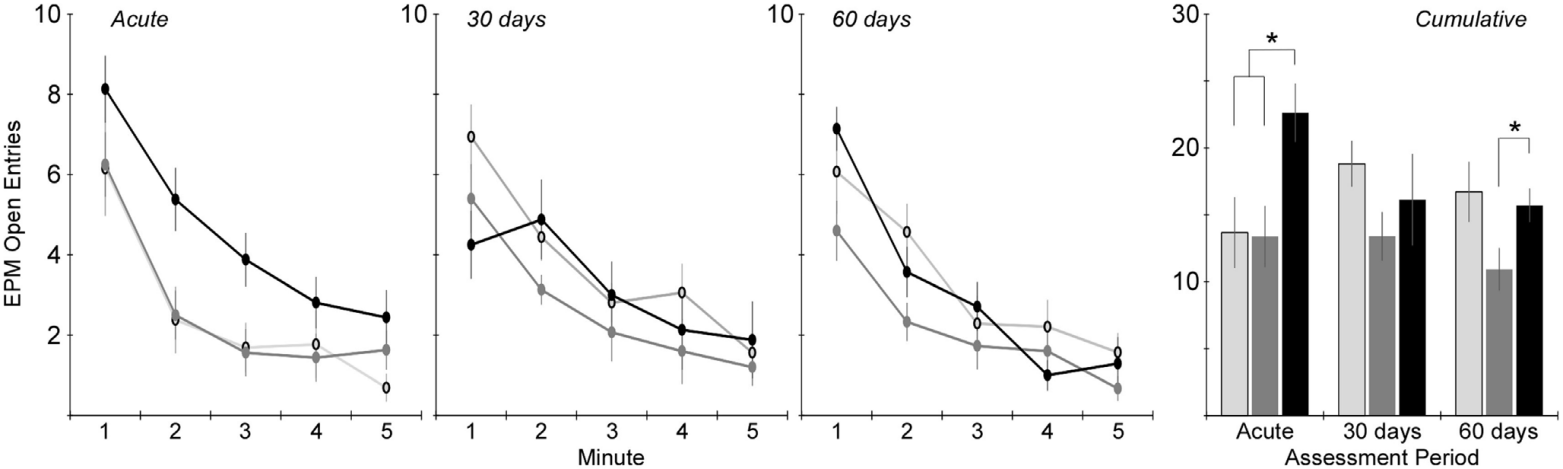
**Total number of entries into the open areas of the elevated plus maze (center + open arms) during the 5-min elevated plus maze protocol (mean ± SE) in control rats (light gray), blast-injured rats (dark gray), and rotationally injured rats (black)**. Minute-by-minute data are presented for each of the assessment time points on the left and cumulative data across all 5 min is presented on the right. Statistically significant pairwise differences during each assessment period are indicated by an asterisk (*).

Changes in anxiety-like behavior for the injured groups were assessed in the EPM by quantifying the number of entries into and the amount of time spent in the open areas of the maze. Two-factor ANOVA revealed that the number of open area entries was significantly dependent on injury group (*p* < 0.005), but not assessment period (Figure 5). *Post hoc* assessments revealed that rats receiving rotational acceleration injury had significantly more open area entries than the other injury groups (*p* < 0.05). Pairwise statistical comparisons with adjustment for multiple comparisons were also conducted to determine statistically significant injury group-based differences at each assessment time. During the acute assessment, the rotational injury group demonstrated a much higher number of open area entries for the (*p* < 0.05) than the blast injury group (+68%) and shams (+65%). The blast injury group demonstrated a trend of decreased open entries compared to the other groups (*p* > 0.05) at the 30-day assessment, and significantly fewer open entries compared to the rotational injury group (*p* < 0.05) at the 60-day assessment.

**Figure 6:**
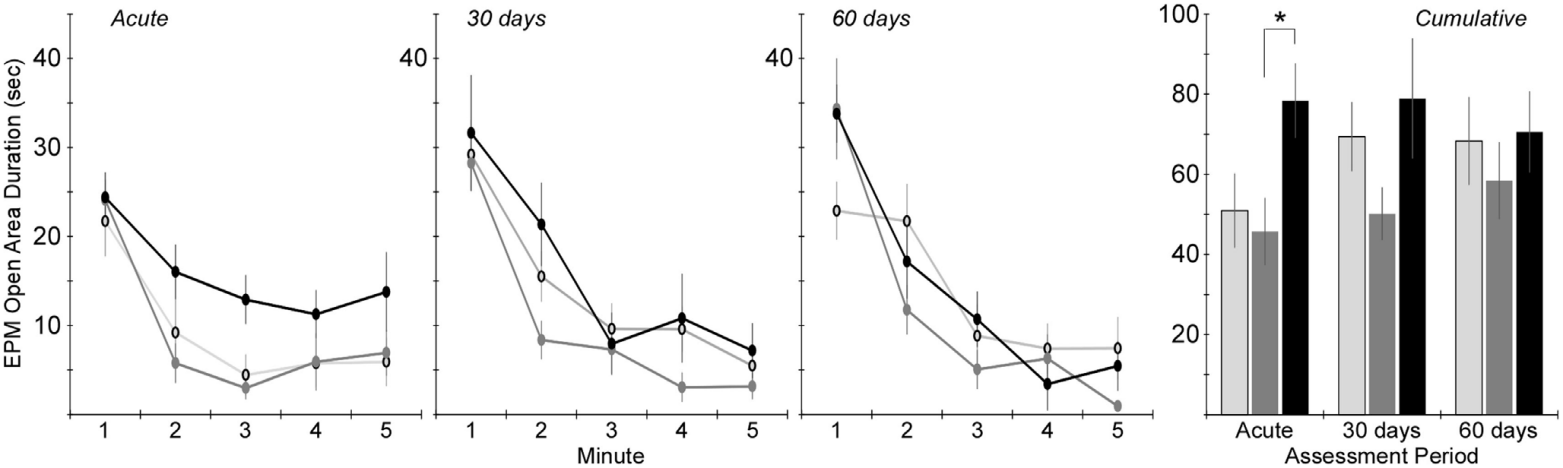
**Amount of time spent in the open areas of the elevated plus maze (center + open arms) during the 5-min elevated plus maze protocol (mean ± SE) in control rats (light gray), blast-injured rats (dark gray), and rotationally injured rats (black)**. Minute-by-minute data are presented for each of the assessment time points on the left and cumulative data across all 5 min is presented on the right. Statistically significant pairwise differences during each assessment period are indicated by an asterisk (*).

Time in the open areas of the EPM demonstrated similar trends (Figure 6). Two-factor ANOVA revealed a significant effect of injury group (*p* < 0.01), but not assessment period. *Post hoc* assessments revealed that rats receiving rotational acceleration injury had significantly more open area time than rats receiving blast injury (*p* < 0.05). Pairwise statistical comparisons with adjustment for multiple comparisons were also conducted to determine statistically significant injury group-based differences at each assessment time. During the acute assessment, rats receiving rotational injury spent significantly more time in the open areas of the maze than rats receiving blast injury. There were no statistically significant injury group-based differences at the 30- and 60-day assessments, although rats receiving blast injury once again demonstrated less time in the open areas of the maze than the other groups during those assessment periods.

Two-factor ANOVA revealed that the duration of time spent in the open areas per entry and open arms per entry was not significantly dependent upon injury group (Table 1), although open area duration per entry was significantly dependent upon assessment period (*p* < 0.05). In general, rats spent more time in the open areas per entry during the 30- and 60-day assessments than during the acute assessment. Additionally, analysis of the amount of time spent in the open areas per entry revealed non-significant injury group-based trends. Both injury groups spent less time in the open areas per entry than controls at the acute assessment. However, that trend reversed at the more chronic time points, with rats receiving rotational injury spending 33% more time per entry than controls at the 30-day assessment and rats receiving blast injury spending 32% more time per entry than controls at the 60-day assessment. Interestingly, injury group-based differences were more evident when analyzing the amount of time spent in the open arms per entry while excluding the center zone. All three groups spent similar time in the open arms per entry during the acute assessment. However, rats receiving rotational injury spent 122% more time per open arm entry than controls and rats receiving blast injury spent 57% more time per open arm entry than controls.

### Morris Water Maze

**Figure 7:**
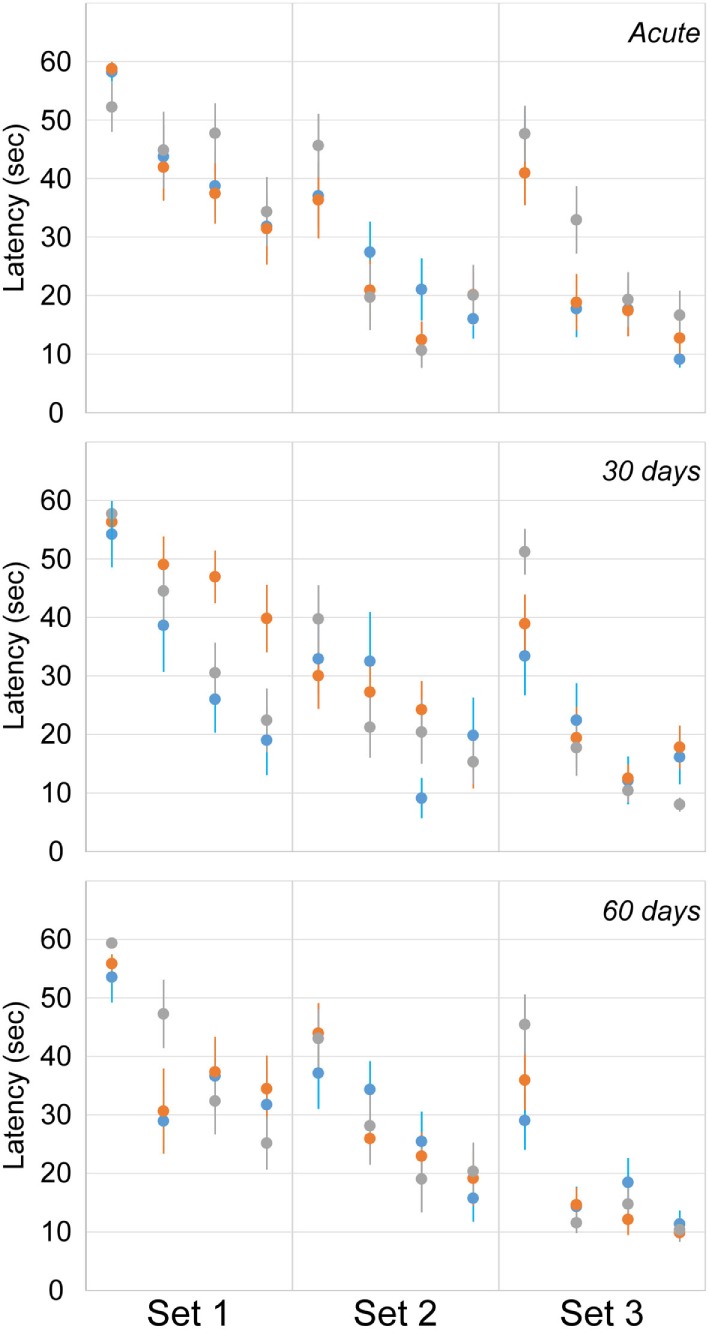
**Latency to find the hidden platform in the Morris water maze (mean ± SE) in control rats (gray), blast-injured rats (orange), and rotationally injured rats (blue)**. Trial-by-trial data are presented for each of the three sets (left to right) at each assessment time points (top to bottom).

Cognitive deficits were assessed using latency to find the hidden platform in the MWM assessment at acute and chronic time points (Figure 7). The number of rats exposed to the MWM at each time point was identical to the EPM (Table 1). A random effect statistical model was used to predict latency and included the fixed effects of trial (1–4), day (1–3), period (acute, 30, and 60), and injury group. Injury group did not have a significant effect on latency to find the hidden platform indicating a lack of significant cognitive deficits for either the blast or rotational injury. Likewise, day and period did not significantly affect latency to find the hidden platform. However, trial number did affect latency to find the hidden platform, indicating the presence of a spatial learning process from trial to trial, across all rats included in this study. However, despite the lack of statistically significant differences in latency, rats receiving blast injury demonstrated 38 and 24% greater mean latency to find the hidden platform compared to the rotational injury and sham groups during the first set of the 30-day assessment, which may indicate some level of cognitive deficit following blast injury.

## Discussion

Mild traumatic brain injury represents a significant clinical problem given patient variability with regard to the severity, duration, and type of symptoms experienced by those afflicted with this condition. This is likely due to current clinical diagnostic criteria for mTBI that do not further distinguish injury beyond severity. This approach fails to consider injury mechanism (e.g., sport impact versus blast exposure) that likely contributes to anatomical or physiological variability. This manuscript presents experimental support that injury mechanism is an important factor that influences the severity, duration, and type of behavioral outcomes following mTBI. The two models presented in this manuscript were designed to reproduce the mechanisms of sports-related concussion (rotational acceleration) and primary blast traumatic brain injury (shock wave overpressure exposure). Adding to the clinical significance of these models is that the biomechanics are biofidelic, repeatable, and scalable to the human. Demonstration of differing outcomes between the two mechanisms can inform clinicians and assist in more accurate diagnoses and mechanism-dependent expectations and treatment protocols. Here, we have presented a more active behavioral phenotype with a lack of inhibition in the EPM that resolves for the most part during the acute phase for rats exposed to sports concussion-type injury. However, blast injury led to an anxiety-like phenotype that manifested into the chronic phase and had stronger evidence of cognitive deficits. Experimental research such as that presented here can continue to outline the role of factors that influence patient outcomes, shaping clinical studies, and eventually contributing to targeted expectations and therapeutics based specifically on characteristics of the concussive event.

The operational relevance of these models is clear. Our laboratory has focused on the development of biomechanically accurate experimental TBI models for over 10 years (26–28), and the current models were designed to replicate distinct injury mechanisms: primary blast and head rotational acceleration. Whereas head rotational acceleration often occurs in isolation in the sporting environment, the military environment presets a more complicated biomechanical scenario, with exposure to blast overpressure (primary blast) often combined with head rotational acceleration (secondary/tertiary blast) (33). However, modeling this complex scenario presents significant experimental issues as each blast exposure presents unique characteristics with regard to overpressure and acceleration magnitudes that can be complicated even more by blunt head impact and polytrauma. For this reason, the present analysis focused on quantifying independent effects of blast overpressure and head rotational acceleration to successfully demonstrate these as separate injury mechanisms. The blast model incorporated head constraint to limit effects of head rotational acceleration during shock wave exposure, placed the animals outside of the blast wind (Figure 2) to limit effects on injury outcomes, and protected the body for a focused blast TBI insult. To that end, measured head accelerations as described in a previous publication from our group (15) were well below the threshold for rotational acceleration-induced injury in the rat according to scaled data from humans and primates (21, 34), and overpressure exposure to the body was insignificant (15), indicating that behavioral deficits from that model were primarily a result of shock wave overpressure exposure. This is an important aspect of the blast model to eliminate confounding effects of other injuries (33), and permits the direct comparison of behavioral changes between primary blast and rotational mechanisms.

Along that line, an important aspect of this comparison was the determination of injury severity equivalence between the two injury mechanisms. Our prior work utilizing these models has demonstrated dose-dependent behavioral and brain structural changes as a function of increasing shock wave overpressure magnitude (15) or head rotational acceleration magnitude and duration (20). Other studies have demonstrated similar dose-dependent outcomes using similar injury models (35). Accordingly, it was decided that injury severity equivalence should be based on recovery time, as loss of consciousness duration is a common clinical marker of concussive injury severity in humans. To that end, rats receiving either blast or rotational injury had similar increases in recovery time (21% for blast and 25% for rotation) compared to their anesthesia protocol-matched controls. It was necessary to compare recovery time in injured rats to their protocol-matched controls as the rotational acceleration protocol incorporated somewhat longer duration of anesthesia due to the placement in the helmet device. This resulted in somewhat longer recovery time for uninjured rotational controls compared to uninjured blast controls. However, these differences were only evident in the very acute phase, as behavioral assessments conducted during the first week or 1–2 months post injury revealed no significant differences between rotational and blast controls. Therefore, control rats from both protocols were grouped for the analysis of behavioral changes in the MWM and EPM assessments.

Significant mechanism-dependent differences in post-injury behaviors were evident between rats receiving rotational injury and rats receiving blast injury. For example, activity-related metrics assessed using the EPM varied in both severity and timing based on injury mechanism. Rats receiving rotational injury tended to have significantly increased activity at the acute assessment, with 81% more arm changes and 65% more open arm entries than controls. Those differences essentially resolved by the 30-day assessment. However, rats receiving blast injury demonstrated no differences in activity from controls at the acute assessment, but had decreased activity at the 30- and 60-day assessments. Those changes also appeared to be progressing, with 27 and 29% decreased arm changes and open entries at the 30-day assessment, and 32 and 35%, respectively, at the 60-day assessment.

Those changes were mirrored by the amount of time spent in the open areas of the EPM. Once again, rats receiving rotational injury spent 54% more time in the center and open arms of the maze at the acute assessment than controls. That difference compared to controls resolved at the 30- and 60-day assessments. Also mirroring activity was that rats receiving blast injury had similar open area time to controls during the acute assessment and decreased (−27%) open area time by the 30-day assessment. Combined, these results suggest that rats that underwent rotational injury had a greater locomotor response to the novel apparatus and were less inhibited by the open areas of the maze in the acute phase after injury, but this disinhibited phenotype resolved by 30 days post injury. Conversely, rats that received blast injury displayed an elevated anxiety-like behavior that was not evident acutely, but emerged at the 30-day time point (36) and remained at 60 days post injury.

These mechanism-dependent behavioral changes were consistent with prior studies incorporating similar injury models. For example, persistent behavioral changes consistent with anxiety were identified in rodents following shock wave exposure (36, 37). Those differences were consistent across multiple behavioral assessments, including the open field test, the elevated zero test, and predator scent exposure. Elevated anxiety-like behaviors in rats following shock wave exposure were attributed to electrophysiological changes (38), mitochondrial distress in the hippocampus (39), neuroinflammation (40), elevated stathmin in the amygdala (36), and elevated levels of corticosterone and protein markers in the hippocampus and prefrontal cortex (41). Far fewer studies have been conducted in rodents incorporating the rotational acceleration-injury mechanism. However, in one study that incorporated rotational acceleration injury (1.5 Mrad/s^2^), rodents demonstrated acute non-significant increases in the percentage of time spent in the open arms of the EPM (42). That study demonstrated increased blood serum levels of neurofilament heavy chain, Tau, and S100B. Therefore, the types of behavioral differences identified in the current study mirror findings from other studies that incorporated similar injury models although specific mechanisms for those changes may require further clarification.

Taken together, our data reveal that rotational and blast injury result in behavioral changes with different qualitative and temporal manifestations. Whereas rotational injury was characterized by a rapidly emerging phenotype consistent with behavioral disinhibition, blast injury was associated with emotional and cognitive differences that were not evident acutely, but developed later, with an anxiety-like phenotype still present at our most chronic measurements.

The differing behavioral outcomes due to blast and rotational injury suggest differing underlying mechanisms of injury, as expected, which could be related to the brain regions affected by the different injury mechanisms. Previous histological and neuroimaging studies have highlighted differences in the manifestation of injury from blast or rotational TBI. In our prior studies performed with identical injury models to those described here, blast shock wave and rotational acceleration exposures caused injury to differing brain anatomical regions as assessed with diffusion tensor imaging (DTI). Blast shock wave exposure was associated with significant groupwise changes in the ipsilateral cortex, medial prefrontal cortex, hippocampus, and thalamus (15). On the other hand, rotational acceleration was associated with alterations in the right internal capsule and corticospinal tract, right amygdala, thalamus, and a portion of the medial cerebral cortex (20). Moreover, the changes in fractional anisotropy (FA), a summary metric derived from DTI, were decreased in blast but in rotational acceleration both significant increases and decreases were observed in differing brain regions. While the changes in FA are somewhat ambiguous with regard to the underlying pathology (43), the results highlight evidence of microstructural changes as opposed to gross structural injuries or hemorrhage. Mild blast and rotational TBI are both known to initiate a complex pathological cascade that includes axonal injury (44), gliosis (45), and inflammation. The precise relationship between injury mechanisms, anatomical location, and behavioral changes warrant further investigation, as understanding the interplay between the brain and behavior is essential from design of protective equipment to planning clinical rehabilitative strategies.

The current study was focused on equating acute injury severity between the two injury mechanisms (shock wave overpressure exposure versus head rotational acceleration) using anesthesia recovery time. However, it should be noted that comparison of recovery time may be complicated by a number of factors including that lesions to specific brain regions are likely to impair recovery times more than other brain regions, total recovery time in this study is a function of both the duration of anesthesia in addition to injury severity, and that the effects of an injury on recovery time is likely different from its effect on behavioral alterations. Nonetheless, this comparison was made for the sake of equating very acute injury severity and not intended to correlate recovery time to specific behavioral alterations. However, on a larger scale, both of the models employed in this study produced very mild levels of traumatic brain injury compared to more commonly used preclinical models that produce significant loss of brain tissue even for “mild” injuries compared to shams in the controlled cortical impact (CCI) model (46, 47) or subdural and intraparenchymal hematoma with brain swelling in the lateral fluid percussion model (48). These pathologies were not present upon gross inspection for the current models and are not commonly associated with the mild injuries often sustained in sporting environments such as American football. Given the consistent lack of significant pathology in the models incorporated in this study in addition to the non-invasive nature of the injury protocols, these models provide additional experimental options for investigation of low severity concussions associated with sporting or military environments, particularly relevant and suited for repetitive injuries or subconcussive exposure.

Injury biomechanics incorporated in this study were generally consistent with prior investigations using similar experimental models. Peak shock wave overpressure is the most common metric reported for studies incorporating shock tube models. Peak overpressures incorporated in this study were considerably higher than prior work (69–236 kPa) (35, 49–51), although Svetlov and colleagues incorporated similar overpressure magnitudes (358 kPa) (52). However, peak overpressure is not the best correlate for injury onset and severity, and an assessment of overpressure duration must be considered (53). Accordingly, shock wave impulse, computed as the area beneath the positive portion of the overpressure versus time pulse, is likely a better indicator of the severity of blast shock wave exposure. Assuming that shock wave profiles fit the Friedlander profile, shock wave impulse can be computed based on peak overpressure and positive duration characteristics. Shorter durations incorporated using the current model (28) result in shock wave impulse values (45 kPa × ms) that are more in line with prior literature. Longer durations produced by the WRAIR model (3–4 ms) (54) and other groups (1–2 ms) (51), combined with lower peak overpressures (116–236 kPa) result in shock wave impulse values between 50 and 216 kPa × ms. Other models incorporating very long durations (10–18 ms) (52, 55) have considerably greater impulse values (640–1,000 kPa × ms) that presumably produce more significant injuries. Mild injuries were produced in mice and organotypic hippocampal slice cultures with shock wave impulse exposures consistent with the current study (31, 32). Therefore, although the peak overpressure metric is somewhat higher in the current model, a more accurate assessment of the severity of shock wave exposure places the current model in line and somewhat on the lower end of a majority of prior work in this area. Fewer studies are available for comparison of biomechanics between rotational acceleration-produced injuries. Davidsson and Risling reported hemorrhage for some rats exposed to sagittal plane head rotational accelerations exceeding 600 krad/s^2^ and β-APP-positive axons for head rotational accelerations above 1,300 krad/s^2^ (44). However, Xiao-Sheng and colleagues reported axonal swelling for coronal plane head rotational accelerations of 204 krad/s^2^ (56). The present study incorporated head rotational accelerations more in line with Xiao-Sheng and colleagues, although our prior work demonstrated a lack of β-APP-positive axons (26). Therefore, the correlation between head rotational acceleration biomechanics and pathological outcomes requires further quantification. Experimental rotational acceleration-injury models will be instrumental to outlining this correlation and understanding the biomechanics of sports concussion.

Although equating injury severity using recovery time as a correlate for future behavioral changes may be somewhat flawed, which was not the intent of this study, the models incorporated were previously shown to produce consistent and dose-dependent behavioral alterations. Therefore, in general, mechanism-dependent behavioral alterations identified in this study are consistent across a variety of injury severities, albeit dose dependent with generally increasing alteration for greater biomechanical exposure. For example, greater activity and more open area time in the EPM was identified following a range of rotational acceleration exposures (20) and less activity and greater closed arm time was identified following multiple shock wave overpressure magnitudes (15). Therefore, the two injury mechanisms incorporated in this study produce different behavioral alterations regardless of acute injury severity. This highlights the importance of these findings in identifying different type and time course of behavioral alterations based on injury mechanism, which may have significant implication with regard to predicting patient outcomes depending on the mechanism of injury. However, results of this admittedly limited study may require further validation and characterization to confirm and more thoroughly outline these differences.

This study demonstrated significant behavioral outcome differences based on the mechanism of mild TBI. Differences were evident in both the severity and type of outcomes, as well as the time course of behavioral changes. Although not thoroughly investigated here, differences are likely attributable to pathologies affecting different brain regions, as evident in our prior MRI studies, or differing mechanisms of tissue damage based on injury model. Rotational acceleration produces inertially modulated strain-based injuries (57) whereas shock wave exposure mechanisms are less clear and have been hypothesized to create tissue damage through blast wave propagation via thoracic mechanisms, ischemic brain damage, head acceleration, direct skull deformation resulting in neuronal damage, or strain-induced tissue damage due to shock wave interaction with brain tissues (7, 12, 15, 33, 35, 53, 58–62). Nonetheless, these findings suggest that mTBI outcomes are dependent upon the mechanism of injury that has both clinical and experimental implications. From a clinical standpoint, along with acute patient condition and personal medical history, details of the injury event may be important in treatment and predicting patient outcomes. From an experimental standpoint, choice of a biomechanically correct injury model is clearly an important aspect of the translational strength of a laboratory-based study. Ongoing research in our laboratory and others will continue to define the importance of injury biomechanics on behavioral, pathological, and imaging outcomes following mTBI.

A possible limitation of the current analysis is the lack of a separate cohort of animals exposed to shock wave overpressure without head constraint. This would permit study of the combined effects of shock wave exposure and head rotational acceleration, a condition more in line with real-world blast exposures. Head rotational acceleration during blast can result from the blast wind and/or head impact from projectiles or falls. A recent experimental study by Goldstein and colleagues investigated this phenomenon with separate cohorts of rodents exposed to shock wave overpressure with and without head immobilization (33). That study reported increased memory and learning deficits when the rodent head was not immobilized and allowed to rotate in the blast wind, suggesting a confounding effect of shock wave overpressure and head rotational acceleration on the resulting injury. However, the magnitude of head rotational acceleration will vary independently of shock wave overpressure magnitude due to a number of factors including velocity of the blast wind, biodynamics of interaction of the blast wind with the head/helmet, and the presence/absence or type of head impact. Therefore, investigation of the confounding effects of shock wave overpressure and head rotational acceleration requires a more comprehensive parametric analysis that accounts for independently varying magnitudes of overpressure and acceleration. This was not the intent of the current study, which was designed to assess the type and time course of behavioral outcomes following two mechanisms of mTBI.

## Author Contributions

Dr. BDS was responsible for study design, analysis of data, interpretation of results, and preparation of manuscript. Mr. AS was responsible for data collection and analysis of results. Dr. MDB was responsible for interpretation of medical imaging data. Dr. CMO was responsible for interpretation of behavioral data. Dr. AG-J was responsible for interpretation of pathology data. Dr. SNK was responsible for clinical applicability of the data. Dr. MM was responsible for translating behavioral findings to the human. Dr. FAP secured funding and was responsible for overall oversight of the project.

## Conflict of Interest Statement

The authors declare that the research was conducted in the absence of any commercial or financial relationships that could be construed as a potential conflict of interest.

## References

[1] Stemper BD Pintar FA. Biomechanics of concussion. Prog Neurol Surg (2014) 28:14–27. 10.1159/000358748 24923389

[2] Young LA Rule GT Bocchieri RT Burns JM. Biophysical mechanisms of traumatic brain injuries. Semin Neurol (2015) 35:5–11. 10.1055/s-0035-1544242 25714862

[3] Sayer NA Chiros CE Sigford B Scott S Clothier B Pickett T Characteristics and rehabilitation outcomes among patients with blast and other injuries sustained during the Global War on Terror. Arch Phys Med Rehabil (2008) 89:163–70. 10.1016/j.apmr.2007.05.025 18164349

[4] Nelson NW Davenport ND Sponheim SR Anderson CR Blast-related mild traumatic brain injury: neuropsychological evaluation and findings. In: Kobeissy FHP, editor. Brain Neurotrauma: Molecular, Neuropsychological, and Rehabilitation Aspects. Boca Raton, FL: CRC Press (2015). 26269927

[5] Lippa SM Pastorek NJ Benge JF Thornton GM. Postconcussive symptoms after blast and nonblast-related mild traumatic brain injuries in Afghanistan and Iraq war veterans. J Int Neuropsychol Soc (2010) 16:856–66. 10.1017/S1355617710000743 20682086

[6] Belanger HG Kretzmer T Yoash-Gantz R Pickett T Tupler LA. Cognitive sequelae of blast-related versus other mechanisms of brain trauma. J Int Neuropsychol Soc (2009) 15:1–8. 10.1017/S1355617708090036 19128523

[7] Moss WC King MJ Blackman EG. Skull flexure from blast waves: a mechanism for brain injury with implications for helmet design. Phys Rev Lett (2009) 103:108702. 10.1103/PhysRevLett.103.108702 19792349

[8] Cernak I Savic J Malicevic Z Zunic G Radosevic P Ivanovic I Involvement of the central nervous system in the general response to pulmonary blast injury. J Trauma (1996) 40:S100–4. 10.1097/00005373-199603001-00023 8606388

[9] Courtney AC Courtney MW. A thoracic mechanism of mild traumatic brain injury due to blast pressure waves. Med Hypotheses (2009) 72:76–83. 10.1016/j.mehy.2008.08.015 18829180

[10] Taylor PA Ludwigsen JS Ford CC. Investigation of blast-induced traumatic brain injury. Brain Inj (2014) 28:879–95. 10.3109/02699052.2014.888478 24766453 PMC4046872

[11] Chen Y Huang W. Non-impact, blast-induced mild TBI and PTSD: concepts and caveats. Brain Inj (2011) 25:641–50. 10.3109/02699052.2011.580313 21604927

[12] Shah AS Stemper BD Yoganandan N Pintar FA Rangarajan N Hallman JJ Methodology to study attenuation of a blast wave through the cranium. ASME International Mechanical Engineering Congress and Exposition. Denver, CO: (2011).

[13] Miller AP Shah AS Aperi BV Budde MD Pintar FA Tarima S Effects of blast overpressure on neurons and glial cells in rat organotypic hippocampal slice cultures. Front Neurol (2015) 6:20. 10.3389/fneur.2015.00020 25729377 PMC4325926

[14] Effgen GB Vogel EW 3rd Lynch KA Lobel A Hue CD Meaney DF Isolated primary blast alters neuronal function with minimal cell death in organotypic hippocampal slice cultures. J Neurotrauma (2014) 31:1202–10. 10.1089/neu.2013.3227 24558968

[15] Budde MD Shah A McCrea M Cullinan WE Pintar FA Stemper BD. Primary blast traumatic brain injury in the rat: relating diffusion tensor imaging and behavior. Front Neurol (2013) 4:154. 10.3389/fneur.2013.00154 24133481 PMC3796287

[16] Margulies SS Thibault LE Gennarelli TA. Physical model simulations of brain injury in the primate. J Biomech (1990) 23:823–36. 10.1016/0021-9290(90)90029-3 2384494

[17] Sabet AA Christoforou E Zatlin B Genin GM Bayly PV. Deformation of the human brain induced by mild angular head acceleration. J Biomech (2008) 41:307–15. 10.1016/j.jbiomech.2007.09.016 17961577 PMC2701725

[18] Li J Zhang J Yoganandan N Pintar F Gennarelli T. Regional brain strains and role of falx in lateral impact-induced head rotational acceleration. Biomed Sci Instrum (2007) 43:24–9. 17487052

[19] Lamy M Baumgartner D Yoganandan N Stemper BD Willinger R. Experimentally validated three-dimensional finite element model of the rat for mild traumatic brain injury. Med Biol Eng Comput (2013) 51:353–65. 10.1007/s11517-012-1004-7 23192366

[20] Stemper BD Shah AS Pintar FA McCrea M Kurpad SN Glavaski-Joksimovic A Head rotational acceleration characteristics influence behavioral and diffusion tensor imaging outcomes following concussion. Ann Biomed Eng (2015) 43(5):1071–88. 10.1007/s10439-014-1171-9 25344352 PMC4654450

[21] Ommaya AK Hirsch AE Tolerances for cerebral concussion from head impact and whiplash in primates. J Biomech (1971) 4:13–21. 10.1016/0021-9290(71)90011-X 5001829

[22] Phillips YY. Primary blast injuries. Ann Emerg Med (1986) 15:1446–50. 10.1016/S0196-0644(86)80940-4 3535591

[23] Guskiewicz KM Marshall SW Bailes J McCrea M Cantu RC Randolph C Association between recurrent concussion and late-life cognitive impairment in retired professional football players. Neurosurgery (2005) 57:719–26. 10.1227/01.NEU.0000175725.75780.DD 16239884

[24] Guskiewicz KM Marshall SW Bailes J McCrea M Harding HP Jr Matthews A Recurrent concussion and risk of depression in retired professional football players. Med Sci Sports Exerc (2007) 39:903–9. 10.1249/mss.0b013e3180383da5 17545878

[25] Tanielian TL Jaycox L Rand Corporation Invisible Wounds of War: Psychological and Cognitive Injuries, Their Consequences, and Services to Assist Recoveryed. Santa Monica, CA: RAND (2008).

[26] Fijalkowski RJ Stemper BD Pintar FA Yoganandan N Crowe MJ Gennarelli TA. New rat model for diffuse brain injury using coronal plane angular acceleration. J Neurotrauma (2007) 24:1387–98. 10.1089/neu.2007.0268 17711400

[27] Fijalkowski RJ Ellingson BM Stemper BD Yoganandan N Gennarelli TA Pintar FA. Interface parameters of impact-induced mild traumatic brain injury. Biomed Sci Instrum (2006) 42:108–13. 16817594

[28] Shah AS Stemper BD Pintar FA. Development and characterization of an open-ended shock tube for the study of blast mtbi. Biomed Sci Instrum (2012) 48:393–400. 22846311

[29] Cheney JA Brown AL Bareyre FM Russ AB Weisser JD Ensinger HA The novel compound LOE 908 attenuates acute neuromotor dysfunction but not cognitive impairment or cortical tissue loss following traumatic brain injury in rats. J Neurotrauma (2000) 17:83–91. 10.1089/neu.2000.17.83 10674760

[30] Saatman KE Contreras PC Smith DH Raghupathi R McDermott KL Fernandez SC Insulin-like growth factor-1 (IGF-1) improves both neurological motor and cognitive outcome following experimental brain injury. Exp Neurol (1997) 147:418–27. 10.1006/exnr.1997.6629 9344566

[31] Vogel EW 3rd Effgen GB Patel TP Meaney DF Bass CR Morrison B 3rd. Isolated primary blast inhibits long-term potentiation in organotypic hippocampal slice cultures. J Neurotrauma (2015). 10.1089/neu.2015.4045 26414012 PMC5583564

[32] Hue CD Cho FS Cao S Nicholls RE Vogel EW 3rd Sibindi C Time course and size of blood-brain barrier opening in a mouse model of blast-induced traumatic brain injury. J Neurotrauma (2015). 10.1089/neu.2015.4067 26414212

[33] Goldstein LE Fisher AM Tagge CA Zhang XL Velisek L Sullivan JA Chronic traumatic encephalopathy in blast-exposed military veterans and a blast neurotrauma mouse model. Sci Transl Med (2012) 4:134ra60 10.1126/scitranslmed.3004862 PMC3739428 22593173

[34] Rowson S Duma SM Beckwith JG Chu JJ Greenwald RM Crisco JJ Rotational head kinematics in football impacts: an injury risk function for concussion. Ann Biomed Eng (2012) 40:1–13. 10.1007/s10439-011-0392-4 22012081 PMC10465647

[35] Long JB Bentley TL Wessner KA Cerone C Sweeney S Bauman RA. Blast overpressure in rats: recreating a battlefield injury in the laboratory. J Neurotrauma (2009) 26:827–40. 10.1089/neu.2008.0748 19397422

[36] Elder GA Dorr NP De Gasperi R Gama Sosa MA Shaughness MC Maudlin-Jeronimo E Blast exposure induces post-traumatic stress disorder-­related traits in a rat model of mild traumatic brain injury. J Neurotrauma (2012) 29:2564–75. 10.1089/neu.2012.2510 22780833 PMC3495123

[37] Cernak I Merkle AC Koliatsos VE Bilik JM Luong QT Mahota TM The pathobiology of blast injuries and blast-induced neurotrauma as identified using a new experimental model of injury in mice. Neurobiol Dis (2011) 41:538–51. 10.1016/j.nbd.2010.10.025 21074615

[38] Park E Eisen R Kinio A Baker AJ. Electrophysiological white matter dysfunction and association with neurobehavioral deficits following low-level primary blast trauma. Neurobiol Dis (2013) 52:150–9. 10.1016/j.nbd.2012.12.002 23238347

[39] Sajja VS Galloway MP Ghoddoussi F Thiruthalinathan D Kepsel A Hay K Blast-induced neurotrauma leads to neurochemical changes and neuronal degeneration in the rat hippocampus. NMR Biomed (2012) 25:1331–9. 10.1002/nbm.2805 22549883

[40] Kovesdi E Kamnaksh A Wingo D Ahmed F Grunberg NE Long JB Acute minocycline treatment mitigates the symptoms of mild blast-induced traumatic brain injury. Front Neurol (2012) 3:111. 10.3389/fneur.2012.00111 22811676 PMC3397312

[41] Kwon SK Kovesdi E Gyorgy AB Wingo D Kamnaksh A Walker J Stress and traumatic brain injury: a behavioral, proteomics, and histological study. Front Neurol (2011) 2:12. 10.3389/fneur.2011.00012 21441982 PMC3057553

[42] Rostami E Davidsson J Ng KC Lu J Gyorgy A Walker J A model for mild traumatic brain injury that induces limited transient memory impairment and increased levels of axon related serum biomarkers. Front Neurol (2012) 3:115. 10.3389/fneur.2012.00115 22837752 PMC3401945

[43] Budde MD Janes L Gold E Turtzo LC Frank JA. The contribution of gliosis to diffusion tensor anisotropy and tractography following traumatic brain injury: validation in the rat using Fourier analysis of stained tissue sections. Brain (2011) 134:2248–60. 10.1093/brain/awr161 21764818 PMC3155707

[44] Davidsson J Risling M. A new model to produce sagittal plane rotational induced diffuse axonal injuries. Front Neurol (2011) 2:41. 10.3389/fneur.2011.00041 21747777 PMC3128930

[45] Svetlov SI Prima V Glushakova O Svetlov A Kirk DR Gutierrez H Neuro-glial and systemic mechanisms of pathological responses in rat models of primary blast overpressure compared to “composite” blast. Front Neurol (2012) 3:15. 10.3389/fneur.2012.00015 22403567 PMC3275793

[46] Washington PM Forcelli PA Wilkins T Zapple DN Parsadanian M Burns MP. The effect of injury severity on behavior: a phenotypic study of cognitive and emotional deficits after mild, moderate, and severe controlled cortical impact injury in mice. J Neurotrauma (2012) 29:2283–96. 10.1089/neu.2012.2456 22642287 PMC3430487

[47] Smith DH Soares HD Pierce JS Perlman KG Saatman KE Meaney DF A model of parasagittal controlled cortical impact in the mouse: cognitive and histopathologic effects. J Neurotrauma (1995) 12:169–78. 10.1089/neu.1995.12.169 7629863

[48] Thompson HJ Lifshitz J Marklund N Grady MS Graham DI Hovda DA Lateral fluid percussion brain injury: a 15-year review and evaluation. J Neurotrauma (2005) 22:42–75. 10.1089/neu.2005.22.42 15665602

[49] Ahlers ST Vasserman-Stokes E Shaughness MC Hall AA Shear DA Chavko M Assessment of the effects of acute and repeated exposure to blast overpressure in rodents: toward a greater understanding of blast and the potential ramifications for injury in humans exposed to blast. Front Neurol (2012) 3:32. 10.3389/fneur.2012.00032 22403572 PMC3293241

[50] Bolander R Mathie B Bir C Ritzel D VandeVord P. Skull flexure as a contributing factor in the mechanism of injury in the rat when exposed to a shock wave. Ann Biomed Eng (2011) 39:2550–9. 10.1007/s10439-011-0343-0 21735320

[51] Risling M Plantman S Angeria M Rostami E Bellander BM Kirkegaard M Mechanisms of blast induced brain injuries, experimental studies in rats. Neuroimage (2011) 54(Suppl 1):S89–97. 10.1016/j.neuroimage.2010.05.031 20493951

[52] Svetlov SI Prima V Kirk DR Gutierrez H Curley KC Hayes RL Morphologic and biochemical characterization of brain injury in a model of controlled blast overpressure exposure. J Trauma (2010) 69:795–804. 10.1097/TA.0b013e3181bbd885 20215974

[53] Bass CR Panzer MB Rafaels KA Wood G Shridharani J Capehart B. Brain injuries from blast. Ann Biomed Eng (2012) 40:185–202. 10.1007/s10439-011-0424-0 22012085

[54] Chavko M Watanabe T Adeeb S Lankasky J Ahlers ST McCarron RM. Relationship between orientation to a blast and pressure wave propagation inside the rat brain. J Neurosci Methods (2011) 195:61–6. 10.1016/j.jneumeth.2010.11.019 21129403

[55] Vandevord PJ Bolander R Sajja VS Hay K Bir CA. Mild neurotrauma indicates a range-specific pressure response to low level shock wave exposure. Ann Biomed Eng (2012) 40:227–36. 10.1007/s10439-011-0420-4 21994066

[56] Xiao-Sheng H Sheng-Yu Y Xiang Z Zhou F Jian-ning Z. Diffuse axonal injury due to lateral head rotation in a rat model. J Neurosurg (2000) 93:626–33. 10.3171/jns.2000.93.4.0626 11014541

[57] Ommaya AK Yarnell P Hirsch AE Scaling of experimental data on cerebral concussion in subhuman primates to concussion threshold for man. 11th Stapp Car Crash Conference Anaheim, CA: (1967). p. 73–80.

[58] Clemedson CJ Blast injury. Physiol Rev (1956) 36:336–54. 13359127 10.1152/physrev.1956.36.3.336

[59] Cernak I Wang Z Jiang J Bian X Savic J. Ultrastructural and functional characteristics of blast injury-induced neurotrauma. J Trauma (2001) 50:695–706. 10.1097/00005373-200104000-00017 11303167

[60] Cernak I. The importance of systemic response in the pathobiology of blast-induced neurotrauma. Front Neurol (2010) 1:151. 10.3389/fneur.2010.00151 21206523 PMC3009449

[61] Courtney MW Courtney AC. Working toward exposure thresholds for blast-induced traumatic brain injury: thoracic and acceleration mechanisms. Neuroimage (2011) 54(Suppl 1):S55–61. 10.1016/j.neuroimage.2010.05.025 20483376

[62] Nakagawa A Manley GT Gean AD Ohtani K Armonda R Tsukamoto A Mechanisms of primary blast-induced traumatic brain injury: insights from shock-wave research. J Neurotrauma (2011) 28:1101–19. 10.1089/neu.2010.1442 21332411

